# Antioxidant and anti-dermatophytic properties leaf and stem bark of *Xylosma longifolium* clos

**DOI:** 10.1186/1472-6882-13-155

**Published:** 2013-07-02

**Authors:** Wangkheirakpam Radhapiyari Devi, S Brojendro Singh, Chingakham B Singh

**Affiliations:** 1 Medicinal and Horticultural Plant Resources Division, Institute of Bioresources and Sustainable Development, Imphal 795001, Manipur, India

**Keywords:** *Xylosma Longifolium*, Phenolic, Flavonoid, Antioxidant, RP-HPLC, Anti-Dermatophytic

## Abstract

**Background:**

The present study was carried out to assess the phytochemical and anti-dermatophytic effect of the leaf and bark extracts of *Xylosma longifolium* Clos. The leaf and stem bark are used by the indigenous people of Manipur, India for treatment of skin diseases.

**Methods:**

The leaves and stem barks of *Xylosma longifolium* were extracted using petroleum ether, chloroform and methanol respectively. The different extracts of each plant parts were tested for antioxidant activity using DPPH assay. The phenolic content was assayed using Folin-Ciocalteu colorimetric method. Each extracts was further analysed by RP-HPLC to quantify some individual flavonoid components. The anti-dermatophytic activity was evaluated both by agar diffusion method and micro wells dilution method against the *Microsporum boullardii* MTCC 6059, *M. canis* (MTCC 2820 and MTCC 32700), *M. gypseum* MTCC 2819, *Trichophyton ajelloi* MTCC 4878, *T. rubrum* (MTCC 296 and MTCC 3272).

**Results:**

The free radical scavenging activity values were ranged from 0.7 to 1.41 mg/ml and 0.6 to 1.23 mg/ml, respectively for leaf and stem bark extracts. The amount of total phenolic contents of the extracts occurred in both leaf and bark in the range of 12 to 56.6 mg GAE/100 g and 16 to 58 mg GAE/100 g respectively. RP-HPLC analysis for flavonoids revealed the presence of two major flavonoid compounds, rutin and catechin. Kaempferol was in trace or absent. Methanol leaf extract showed significant low inhibitory effect against tested fungus *Trichophyton ajelloi* MTCC 4878 (0.140625 mg/ml) as the most sensitive. These finding suggest that the methanol leaf extract tested contain compounds with antimicrobial properties.

**Conclusion:**

The results of our study may partially justify the folkloric uses on the plant studied and further provide an evidence that the leaf extract of *Xylosma longifolium* might be indeed a potential sources of antimicrobial agents.

## Background

Medicinal plants are known to be sourced of diverse nutrients and non-nutrient molecules. Many of the medicinal plants display antioxidant and antimicrobial properties which can protect the human body against both cellular oxidation reactions and pathogens. Antioxidants are vital substances which possess the ability to protect the body from damage caused by free radical induced oxidative stress [1]. There is an increasing interest in natural antioxidants, e.g., polyphenols, present in medicinal and dietary plants, which might help to prevent oxidative damage [2]. The presence of phenolic compounds (phenolic acids, polyphenols and flavonoids) in plants, herbs and spices, is gaining increasing attention because of their various functions, such as antioxidant activity and health benefits [3]. Thus, it is important to characterize different types of medicinal plants for their antioxidant and antimicrobial potentiality [4-6]. Medicinal plants are known to produce certain bioactive molecules which are responsible for their antimicrobial properties [7-10]. The substances that can inhibit pathogens and have little toxicity to host cells are considered as candidates for developing new antimicrobial drugs.

The incidence of dermatophytic infections has increased considerably during the past several decades [11]. Dermatophytes are responsible for serious human pathogenic disorders in various parts of the world. Although control measures are available, they have limited effectiveness. Conventional antifungal agents such as chlorohexidine and imidazole derivatives have limited uses. Due to their common side effects such as hepatotoxicity, nausea, diarrhoea and impotency [12], the use is restricted in pregnant and the young people.

In order to identify and develop the novel antimicrobial agent, a number of essential oils and extracts have been investigated extensively to achieve higher levels of human safety standards [13,14]. The screening of such natural products may offer potential resources since their use is widespread.

*Xylosma longifolium* Clos. (Flacourtiaceae) is a medium sized tree, distributed in subtropical Himalaya, North-East India and East to West China. In India, *X. longifolium* is grown for its edible fruits [15], used in Assam, for intoxication, and also exhibits antispasmodic, dysentery, restlessness and insomnia [15,16] and also in Manipur, the fresh leaf and stem bark extracts is also used for curing ringworm, scabies and acne in folk medicine [17].

Medicinal substances from plant species of genus *Xylosma* exhibit antispasmodic, narcotic, and sedative [18] and against spider bites [19]. Methanol leaf, stem and root of *X. terrae-reginae* show antioxidant activity (IC_50_) from 10, 9.7 and 10.5 μg/ml respectively [20]. The root extract of *X. terrae-reginae*, the fractions of the leaves and barks of the roots of *X. ciliatifolium* and methanol leaf extract *X. congestum* result antibacterial activity [20-23]. Two new glucosides, 3-methoxy-4-hydroxyphenylpropane-7, 8-(6′-benzoyl-2′, 1′-O-β-glucopyranosyl)-7, 8, 9-trio, and 2-hydroxyphenyl-4-caffeoyl-β-d-glucoside, together with seven known compounds are isolated from the stem bark of *X. longifolia* and 8-hydroxy-6-methoxy-pentylisocoumarin exhibits against *Mycobacterium tuberculosis*[24]. The methanolic extract of the leaves of *X. longifolium* isolate β-sitosterol, β-amyrin, friedelin, olean-12-en-3α-ol-28-oic acid 3α-D-glucopyranoside [25], flavonoid kaempferol-3-β-xylopyranoside-4’-α-rhamnoside [26]. The aqueous and alcoholic leaf extracts of *X.longifolium* was investigated against bacterial and fungal strains [26].

There is no information available regarding the utilization of *X. longifolium* as a source of anti-dermatophytes. Therefore, the present objectives of this study are to evaluate its leaf and barks extracts for anti-dermatophytic properties which is used in folk medicine in Manipur, India as well as to find its chemical profiles such as total phenol, antioxidants and its flavonoids using three organic solvents with different polarity (petroleum ether, chloroform and methanol) for extraction.

## Methods

### Plant materials

The stem barks and leaves *X. longifolium* were collected from Imphal, Manipur, India. The plant material was identified by Botanical Survey of India, Kolkata and the herbarium voucher sample (WR234) was deposited in the Departmental Herbarium.

### Plant extract

500 g of shade-dried pulverized plant materials were subjected to extraction in a Soxhlet apparatus successively with petroleum ether, chloroform and methanol, the volume of each solvent was 6 times the volume of plant extract. For each solvent, extraction was conducted until no more coloured matter was extracted. Solvent from each extracted mixture was evaporated to dryness using a rotary evaporator under reduce pressure at 40°C. All dried extracts were then kept in tightly fitting stopper bottles and stored in −4°C.

### Determination of total phenolic contents

The total phenolic content (TPC) in the extracts was determined by the Folin-Ciocalteu colorimetric method given by Singleton & Rossi [27]. Different concentrations of extracts were mixed with 1.0 ml of 10 fold diluted Folin-Ciocalteu reagent and 1 ml of saturated sodium carbonate solution. After allowing it to stand for 30 min at 30°C, the absorbance was measured at 725 nm with a UV-Visible spectrophotometer (Multiskan Spectrum, Thermo Scientific). Standard curve of gallic acid solution (10, 20, 40, 60, 80 and 100 ppm) was prepared using the similar procedure. Total phenolic was calculated using a standard gallic acid curve and results were expressed as mg gallic acid equivalent (GAE)/100 g of extract.

### DPPH radical scavenging assay

The scavenging activity of extracts on DPPH was determined using the method described by [28]. This method depends on the reduction of purple DPPH to a yellow coloured diphenyl picrylhydrazine. The determination of the disappearance of free radicals was done using UV-visible spectrometer (Multiskan Spectrum, Thermo Scientific). The remaining DPPH which showed maximum absorption at 517 nm was measured. Each plant extract sample’s stock solution (1.0 mg/ml) was diluted to final concentrations of (0.6 .0.5, 0.4, 0.3, 0.2, 0.1, 0.05 and 0.01) mg/ml, in ethanol. One ml of a 0.3 mM DPPH ethanol solution was added to 2.5 ml of sample solution of different concentrations. These are test solutions. One ml of ethanol was added to 2.5 ml of sample solution of different concentration. These are blank solutions. One ml DPPH solution plus 2.5 ml of ethanol was used as a negative control. The blank for this solution is ethanol. As DPPH is sensitive to light, it is exposed to the minimum possible light. These solutions were allowed to react at room temperature for 30 minutes. The absorbance values were measured and converted into the percentage antioxidant activity using the following equation:

$$\mathrm{Scavenging}\;\mathrm{capacity}\left(\%\right)=100–\left[\begin{array}{l} \left(\text{absorbance of sample–absorbance of blank}\right)\\ \times 100/\text{absorbance of control} \end{array}\right]$$

The tests were done in triplicate. The SCa50 values were calculated by linear regression of plots, where the abscissa represents the concentration of the tested plant extracts and the ordinate the average percent of scavenging capacity. The concentration of sample required to scavenge 50% of DPPH (SCa50) were determined.

### High performance liquid chromatography (HPLC) analysis

The flavonoid compounds present in the polar solvents can be separated by using Reverse Phase HPLC equipped with a photo-diode array detector (RP-HPLC-PDA). This establishes the light absorbance spectrum from visible and UV wavelengths of each detected compound, and show intense absorption in the UV region of the spectrum. Identification of the flavonoid compounds (Rutin, Catechin, Kaempferol) present in the extracts of leaves and barks of *X. longifolium* was performed on a Waters HPLC system equipped with reversed phase X-bridge™ C18 (4.6 mm × 250 mm i.d. 5 μm) column (Waters). The column temperature was set at 26°C [29]. Using a binary pump (Waters, Model No. 1525) on a binary solvent system consisting of (A) acetonitrile and (B) water in the ratio of (80:20 v/v) as mobile phase in isocratic mode with a constant flow rate of 1 ml/min. this was monitored at an absorbance of 259, 280 and 360 nm each of Rutin [30,31], Catechin [30,32] and Kaempferol [33] respectively, using a UV-detector (Photo-Diode Array, Waters Model No. 2996). The sample injection volume was 20 μl. The compounds were identified by comparing with standards of each identified compound using the retention time, the absorbance spectrum profile and also by running the samples after the addition of pure standards. The concentration of an individual flavonoid compounds (Rutin, Catechin, Kaempferol) in the plant extract was calculated based on peak height measurement and then finally converted to mg flavonoid/100 g DW.

### Preparation of standard solutions

The standard solutions were prepared by dissolving each of the standards (namely Rutin, Catechin, Kaempferol) separately in methanol (HPLC grade) using serial dilutions. 10ppm was chosen as the optimum concentration which was within the detection range.

### Microorganisms

The human skin infectious fungal pathogens used in the study (*Microsporum boullardii* 6059, *M. canis* (2820 and 3270), *M. gypseum* 2819, *Trichophyton ajelloi* 4878, *T. rubrum* (296 and 3272) were collected from Microbial Tissue Collection Culture (MTCC), Chandigarh, India. All the strains are maintained on sabouraud dextrose agar (SDA, Hi-Media) at 4°C.

### Preparation of the spore suspension and test sample

The fungi were grown on SDA plates at 32±2°C for 7–9 days, after which the spores were harvested from sporulation colonies and suspended in sterile normal saline water (0.9%). The turbidity of the resulting suspension was compared to 0.5 McFarland turbidity standards. The level of turbidity was equivalent to approximately 1.0×10^8^ spores/ml [34,35].

### In vitro anti-dermatophytic susceptibility assay

#### Disc diffusion assay

The *in vitro* anti-dermatophytic activity of the extracts was assessed by agar well diffusion method using SDA in 10 cm petri dishes [36]. Six wells were pierced using sterile 6mm diameter cork borer in the agar, equidistant, and away from the border. Different concentrations of the extracts (9, 4.5, 2.25, 1.125 mg/ml) were prepared by two-fold dilution method. The graded concentrations of the extracts (20 μl) were deposited into the wells using micropipettes and left for 10 min at room temperature for diffusion. Negative and positive control was prepared using DMSO (final concentration of the DMSO in the highest concentration of plant extract tested did not exceed 0.4% (v/v) and did not affect the cell proliferation) and Amphotericin-B (16 μg/ml), respectively. The plates were inoculated at 32±2°C for 7–9 days. The experiment was repeated thrice and the average results were recorded in radial (mm).

#### Determination of minimum inhibitory concentration

Minimum inhibitory concentrations were determined using the micro wells dilution method [37]. The minimum inhibitory concentrations (MICs) of the crude extracts were determined by two fold serial dilution against the pathogens. Six microliter samples of tested extracts were dissolved in the DMSO (99.5%) employed to isolate and extract the crude extracts. These solutions were serially diluted with the DMSO and were added to sabouraud dextrose broth (SDB) at final concentrations of 0.0703125, 0.140625, 0.28125, 0.5625, 1.125, 2.25, 4.5, 9 mg/ml. A 10 μl spore’s suspension (1.0×10^8^ spores/ml) of each test pathogens was inoculated in the test tubes in SDB medium and incubated at 32±2°C for 2–7 days. The control tubes containing SDB medium were inoculated only with fungal spore suspension. The minimum concentrations at which no visible growth were observed was defined as the MICs, which were expressed in mg/ml.

### Statistical analysis

Sampling proceeds on three independent replications (n=3) for each analytical parameters. Results presented in tables were reported as means ± standard deviation (SD). Data were subjected to one-way analysis of variance ANOVA, and the significant difference between means was determined by Duncan’s multiple range test. Differences at *P* < 0.05 were considered statistically significant. Coefficients of determination (*r*^*2*^) were calculated using Microsoft Excel 2010.

## Results

### TPC and DPPH scavenging activity

The total phenolic contents of the extracts from both leaf and bark (petroleum ether, chloroform and methanol) of *X. longifolium* were tested, and occurred in the range of 12±1.27 to 56.6±4.84 mg GAE/100 g and 16± 1.2 to 58±2.25 mg GAE/100g respectively (Table 1). The total phenolic content of various leaf and bark extracts of petroleum ether, chloroform and methanol were noted to be 12±1.27, 34.6±2.04 and 56.6±4.84 mg GAE/100g and 16±1.2, 37.6±2.1 and 58±2.25 mg GAE/100 g of dry extract, respectively.

**Table 1:** **The total phenolic, its flavonoid and SCa_50_ of *Xylosma longifolium *extracts**

**Type of extract**		**Total phenolic content (mg/100 g)**	**SCa**_**50**_**(mg/ml)**	**Individual flavonoids (%)**		
				**Rutin**	**Catechin**	**Kaempferol**
Leaf	PE	12±1.27^b^	0.7±0.2^a^	0.67	4.29	0
	CH	34.6±2.4^d^	1.3±0.12^a^	0.65	1.73	0.03
	ME	56.6±4.84^h^	1.4±0.04^a^	0.56	1.24	0.08
Bark	PE	16±1.2^b^	0.6±0.17^a^	0.51	1.88	0
	CH	37.6±1.94^c^	1.0±0.35^a^	0.22	1.56	0.021
	ME	58±2.25^d^	1.23±0.56^b^	0.12	1.72	0.07

PE – petroleum ether, CH - chloroform, ME – methanol.

Means ± SD of triplicates. For each column, values followed by one or more of the same letters were not significant different at *P* < 0.05.

The DPPH free radical scavenging activity of the both leaf and bark extracts (petroleum ether, chloroform and methanol) of *X. longifolium* was studied (Table 1). The SCa_50_ values of both leaf and bark extracts were recorded in the range of 0.7±0.2 to 1.4±0.04 mg/ml and 0.6±0.17 to 1.23±0.56 mg/ml, respectively.

### Flavonoid compounds analysis

The solvent extracts were analysed by HPLC to quantify the selected parts of the plant. The established HPLC-UV method was successfully applied for the determination of the three major flavonoid compounds (Rutin, Catechin, Kaempferol) present in different parts of the plant, *X. longifolium*. The contents of these compounds distributed in different parts of the plant, bark and leaf were shown in Table 1. It can be seen that these compounds showed significant variations between the plants parts tested. The content of Rutin in leaf extract was found to be higher in petroleum ether (0.51%) than in chloroform (0.22%) and methanol extracts (0.12%) whereas in bark extract was resulted to be higher in petroleum ether (0.67%) than in chloroform (0.65%) and methanol extracts (0.56%) (Table 1). The content of Catechin in leaf extract was found to be higher in petroleum ether extract (1.88%) than in methanol (1.72%) and chloroform (1.56%) whereas in bark extract was studied to be higher in petroleum ether extract (4.29%) than in chloroform (1.73%) and methanol extract (1.24%) (Table 1). However, the contents of Rutin and Catechin presence found to be more in bark extracts than the leaf extracts. From the Kaempferol content studies it was in trace or absent.

### Relation among DPPH scavenging activity, total Phenolic, and its flavonoid compounds

According to the results of the current study correlation between the content of either total phenolic or individual flavonoids and DPPH scavenging activity was calculated. There were a positive linear relationship between total phenolic content and DPPH scavenging activity of both leaf and bark extracts (*r*^2^ = 0.86 and *r*^2^ = 0.98 respectively). A highly significant correlation was found in bark extracts between total phenolic content and DPPH scavenging activity. The correlation with catechin and rutin in leaf and bark extracts were *r*^2^ = 0.99 and *r*^2^ = 0.54; *r*^2^ = 0.39 and *r*^2^ = 0.98 respectively. Thus rutin in leaf and catechin in bark extracts were found to be decreased.

### Anti-dermatophytic activity and MIC of extracts

The extracts exhibited a moderate anti-dermatophytic activity against the tested fungal pathogens. As shown in Table 2, different solvent extracts from leaf (9 mg/well) showed potent inhibitory effect on the growth of *M. canis* 3270 and *M. gypseum* 2819 (13 mm in radial) by petroleum ether; *M. canis* 3270 (14 mm in radial) by chloroform and *T. ajelloi* 4878 (19 mm in radial) by methanol. Again, different solvent extracts from bark exhibited the efficacy on the inhibition by petroleum ether of *M. canis* 3270 (15 mm in radial), by chloroform of *M. gypseum* 2819 (14 mm in radial) and by methanol of *T. ajelloi* 4878 (15 mm in radial) against all the fungal pathogens tested (Table 2). Thus comparative evaluation of the extracts showed variation in the levels of activity against the tested fungal strains (Table 2). This variation of susceptibility from one species to another is evident from their zones of inhibition. In general, *M. canis* 3270 and *T. ajelloi* 4878 were found to be the most susceptible organisms to both methanol extracts of leaf and bark and requires chemical characterization for its bioactive principle. The diluted concentration of each extracts (4.5, 2.25, 1.125 mg/ml) did not show anti-dermatophytic activity.

**Table 2:** **Anti-dermatophytic activity (zone of inhibition and MIC) of *Xylosma longifolium *extracts compared with commercial antibiotic 16 μg/ml Amphotericin-B**

**Part of plant**	**Extracts**	**Anti-dermatophytic activity**	**MB**	**MC-1**	**MC-2**	**MG**	**TA**	**TR-1**	**TR-2**
Leaf	PE	A	-	10±1.3^c^	13±1.4^c^	13±1.6^d^	10±1.4^c^	-	-
		B	>9	2.25	1.125	1.125	2.25	>9	>9
	CH	A	10±1.6^d^	10±1.3^c^	14±1.6^d^	-	11±1.2^c^	-	-
		B	2.25	2.25	0.562	>9	2.25	>9	>9
	ME	A	10±1.2^c^	13±1.3^c^	14±1.3^c^	-	19±1.5^c^	-	10±1.1^c^
		B	2.25	1.125	2.25	>9	0.140625	>9	2.25
Bark	PE	A	-	-	15±1.2^c^	10±1.4^c^	12±1.2^c^	10±1.4^c^	10±1.4^c^
		B	>9	>9	0.5625	2.25	2.25	2.25	2.25
	CH	A	-	-	10±1.3^c^	14±1.4^c^	12±1.5^c^	11±1.3^c^	12±1.4^c^
		B	>9	>9	2.25	0.5625	2.25	2.25	1.125
	ME	A	-	-	13±1.2^c^	13±1.2^c^	15±1.4^c^	12±1.2^c^	14±1.3^c^
		B	>9	>9	2.25	2.25	0.28125	2.25	1.125
Amphotericin-B		A	16±0.9^b^	17±1.0^b^	19±1.0^b^	19±0.8^b^	20±1.1^c^	16±0.9^b^	18±1.1^c^
		B	1	0.125	0.06	0.25	<0.06	0.25	0.125
DMSO		-	-	-	-	-	-	-	

A –The values (average of triplicate) are radial of zone of inhibition at 9 mg/ml; B – MIC (mg/ml) mean value n = 3, P < 0.05.

PE – Petroleum ether; CH – Chloroform; ME - Methanol.

*Microsporum boullardii* MTCC 6059 - MB; *M. canis* MTCC 2820 - MC-1; *M. canis* MTCC 3270 - MC-2; *M. gypseum* MTCC 2819 - MG; *Trichophyton ajelloi* MTCC 4878 -TA; *T. rubrum* MTCC 296 - TR-1; *T. rubrum* MTCC 3272 - TR-2; – no activity.

Means ± SD of triplicates. For each column, values followed by one or more of the same letters were not significant different at *P* < 0.05.

According to the results (Table 2), the MICs of different leaf extracts on *M. boullardii* 6059, *M. canis* 2820, *M. canis* 3270, *M. gypseum* 2819, *T. ajelloi* 4878, *T. rubrum* 296, *T. rubrum* 3272 were found to be 2.25, 2.25, 0.5625, 1.125, 0.140625, >9, >9 mg/ml, respectively. *M. canis* 3270 and *T. ajelloi* 4878 were found to be the most susceptible fungal pathogens to the chloroform and methanol leaf extracts of *X. longifolium*, respectively. Also the different dark extracts displayed potential effect of anti-dermatophytic activity as minimum inhibitory concentrations against the tested fungal pathogens, with their respective MIC values of *M. boullardii* 6059, *M. canis* 2820, *M. canis* 3270, *M. gypseum* 2819, *T. ajelloi* 4878, *T. rubrum* 296, *T. rubrum* 3272 were found to be >9, >9, 0.5625, 2.25, 0.28125, 2.25, 2.25 mg/ml, respectively. As control, the DMSO did not affect the growth of the sample strains at the concentration used in this study. However, low to moderate anti-dermatophytic effect (9–0.140625 mg/ml) of bark extracts was observed against the tested fungal pathogens as a minimum inhibitory concentration. Highest sensitivity was shown by the entire test microbial towards leaf methanol extract against fungus *T. ajelloi* MTCC 4878 (MIC= 0.140625 mg/ml). The values of the plant extracts were compared with that of the reference antifungal drug Amphotericin-B (Table 2).

## Discussions

In this study, the extraction of both leaf and bark was performed using three solvents of increasing polarity. Different solvent systems have been used for extractions from plant material [38] because the extraction yield is dependent on the polarity and the nature of solvents [39]. Trabelsi *et al.*[40] showed that the methanol is the best solvent to extract total phenolics. The ANOVA for all parameters assessed a significant influence on phenolic contents, antioxidant and antimicrobial activities of plant extracts. ANOVA is just a material used to look for significant differences.

The antioxidant activity of the extracts was determined using a DPPH scavenging assay. The DPPH assay is often used to evaluate the ability of antioxidants to scavenge free radicals which are known to be a major factor in biological damages caused by oxidative stress. This assay is known to give reliable information concerning the antioxidant ability of the tested compounds [41]. The principle of the assay is based on the colour change of the DPPH solution from purple to yellow as the radical is quenched by the antioxidant [42].

These results were consistent with the findings of many research groups who reported such correlation between total phenolic content and SCa_50_ antioxidant activity [43-46]. The Folin–Ciocalteu assay used herein provides a crude estimation of the total phenolic compounds present in the extracts. Since it is not specific to polyphenols any interfering compounds may react with the reagent and thus leads to noticeable elevated phenolic concentrations [47]. In addition, phenolic compounds respond differently in this assay when the number of phenolic groups varies [27]. Several studies showed good correlation between total phenols and antioxidant activity [42,48-50].

Correlation between the content of either total phenol or individual flavonoids and antioxidant capacity data indicate that the antioxidant activity was obtained directly from phenolics, and also particularly catechin and rutin, and assures other experiments that mentioned the same vital role of catechin and rutin in lipid peroxidation inhibition [51,52].

In the present study, methanol leaf and bark extracts showed high antioxidant activities as compared to the other crude extracts. This is due to bioactive compounds such as polyphenols including tannins, flavonoid existed in high polar extracts [53]. Polyphenols are one of the major plant compounds with antioxidant activity. The antioxidant activity of phenolic compounds is reported to be mainly due to their redox properties [54], which can play an important role in adsorbing and neutralizing free radicals, quenching singlet and triplet oxygen, or decomposing peroxides. Organic extracts may be more beneficial than isolated constituents, because other compounds present in the extracts can change the chemical or biological properties of bioactive individual component [55].

Phenolic was also found to be one of the most constituents in both methanol leaf and bark extracts of *X. longifolium*. These may be due to the presence of high bioactive compounds as compared to other organic extracts. The key role of phenolic compounds to scavenge free radicals has been emphasized in several reports [56-62]. The phenolic compounds may contribute directly to the antioxidant action [63]. Total phenol content in the leaf and bark are comparable but varies with the extracting solvent, methanol been the best extracting solvent, follow by chloroform and petroleum ether.

RP-HPLC analysis is the most widely used method for the identification of plant flavonoid compounds. Because of the diversity and complexity of natural flavonoid in medicinal plants, it is difficult to characterize every compound and elucidate its structure. In *X. longifolium* leaf and bark extracts of petroleum ether result the presence of high flavonoids than other extracts. Flavonoid compounds can be defined as a large series of chemical constituents possessing at least one aromatic ring, bearing hydroxyl and other sub-constituents [64]. Increasingly, flavonoids are becoming the subject of medical research. They have been reported to possess many useful properties, including anti-inflammatory activity, oestrogenic activity, enzyme inhibition, antimicrobial activity, anti-allergic activity, antioxidant activity, vascular activity and cytotoxic anti-tumour activity [26,65-71]. For centuries, preparations that contain flavonoids as the principal physiologically active constituents, have been used by physicians and lay healers, in attempts to treat human diseases [65]. Owing to the widespread ability of flavonoids to inhibit spore germination of plant pathogens, they have been proposed for use against fungal pathogens in man [66].

Natural products may constitute an appropriate source of anti-infective agents. For example, flavonoids showed antimicrobial activity [69]. In fact, rutin displayed various biological activities that are beneficial to human health [72]. Studies showed that rutin give high positive correlation with DPPH in bark extracts. This is in agreement with previous study [73] which reported that the antimicrobial spectrums of the catechins. Catechin is the most represented individual flavonoid in all extracts and is best extracted by petroleum ether. The higher concentration is found in leaf.

There is a constant striving to develop safe and new natural antifungal agent to cure the fungal disorders of the increasing social and health implications, where the dermatophytes estimated lifetime risk of acquiring infection. Methods for less deleterious side effects of treatments include use of natural antifungal agents from essential oils and extracts than synthetic drugs [74].

In general, extracts derived from plants are considered as non-phytotoxic compounds and potentially effective against several microorganisms including many fungal and bacterial pathogens [75,76]. Since ancient times, interest have been given on the development of safer antifungal agents to control severe fungal diseases by the extracts and essential oils [76-78]. The results of the antifungal screening showed that the methanol leaf extract of *X. longifolium* have potential anti-dermatophytic activity against some of the skin fungal pathogens. This might be due to the presence of several bioactive compounds in extract of *X. longifolium* as evident by the finding of others [20-26,74-79]. Globally, the anti-dermatophytic activities of all the extracts are very low and seem not to be directly link to their total phenol and individual flavonoids contents. Besides, millions of people throughout the world are affected by superficial fungal infections, which are the most common skin diseases. These infections, which occur in both healthy and immune persons, are caused mainly by dermatophytes. In this study, it was observed that both leaves and barks methanol extracts of *X. longifolium* have great potential to inhibit the *T. ajelloi* 4878, causing superficial fungal infections of the skin known as tinea infections.

Antimicrobial activities observed in this study might be due to the presence of flavonoid compounds or various other bioactive compounds. Extracts from various medicinal plants containing phenolic and flavonoids compounds have been previously reported to possess antimicrobial activity [80,81]. The properties of gallic, caffeic acids, vanillic acid, rutin, and quercetin of different wine were investigated against pathogenic microorganisms [82]. The presence of these compounds might contribute to antimicrobial activity [20,22-26,77]. The flavonoid compounds have antimicrobial activity against human pathogenic microorganisms with some mechanisms of action such as inhibition of nucleic acid synthesis, cytoplasmic membrane function and energy metabolisms. The antimicrobial activity of the extracts of *X. longifolium* might be due to one of such mechanisms of action mentioned above.

The results of this study suggested that *X. longifolium* extracts can be used against many tinea infections such as *Epidermophyton* sp., *Microsporum* sp. and *Trichophyton* sp. where the pathogens have developed resistance against the specific fungicides [24,26,83,84]. The development of natural antifungal agents would also help to decrease the negative impact of synthetic agents such as residues, resistance and environmental pollution.

## Conclusions

The results of our study may partially justify the folkloric uses of this plant in traditional medicine and further provide an evidence that the leaf extract of *Xylosma longifolium* might be indeed a potential sources of antimicrobial agents as well as natural antioxidants. Further studies are required to isolate and characterize the bioactive molecules present in organic extracts of *X. longifolium* to evaluate proper action mechanism of the observed activities.

## Abbreviations

DMSO: Dimethyl sulfoxide; DPPH: 1,1-Diphenyl-2-Picrylhydrazyl; DW: Distilled water.

## Competing interests

The author(s) declare that they have no competing interests.

## Authors’ contributions

RDW carried out the major portion of the work while BSS contribute in the HPLC part. BSC supervised the overall work. All authors the proofread and approved the final manuscript.

## Pre-publication history

The pre-publication history for this paper can be accessed here:

http://www.biomedcentral.com/1472-6882/13/155/prepub
